# Highly selective ethanol gas sensor based on CdS/Ti_3_C_2_T_*x*_ MXene composites†

**DOI:** 10.1039/d4na00927d

**Published:** 2025-01-08

**Authors:** Ly Tan Nhiem, Jianbin Mao, Qui Thanh Hoai Ta, Soonmin Seo

**Affiliations:** a Faculty of Chemical and Food Technology, Ho Chi Minh City University of Technology and Education 01 Vo Van Ngan Street, Linh Chieu Ward, Thu Duc City Ho Chi Minh City Vietnam nhiemlt@hcmute.edu.vn; b College of BioNano Technology, Gachon University Gyeonggi 13120 Republic of Korea mg2895852@gmail.com soonmseo@gachon.ac.kr; c Institute of Chemical Technology, Vietnam Academy of Science and Technology 1A TL29 Street, Thanh Loc Ward, District 12 Ho Chi Minh City 700000 Vietnam tathanhhoaiqui2292@gmail.com

## Abstract

Sensing of hazardous gases has an important role in ensuring safety in a variety of industries as well as environments. Mainly produced by the combustion of fossil fuels and other organic matter, ethanol is a dangerous gas that endangers human health and the environment. Stability and sensing sensitivity are major considerations when designing gas sensors. Here, a superior ethanol sensor with a high response and fast recovery was synthesized by “wrapping” CdS nanoparticles on metallic Ti_3_C_2_T_*x*_ MXene using a simple method. CdS nanoparticles were uniformly covered on the Ti_3_C_2_T_*x*_ MXene surface, forming a “rice crust”-like heterostructure. The sensor displayed good detection of ethanol gas at room temperature. Response signals up to 31% were obtained for ethanol molecules (20 ppm) with quick recovery (41 s). The performance of the ethanol sensor was evaluated across a range of concentrations (5–100 ppm) and relative humidity (60% and 90% RH) at room temperature. Our method could open up a new strategy for the development of ethanol sensors.

## Abstract 2

Sensing of hazardous gases has an important role in ensuring safety in a variety of industries as well as environments.

## Graphical abstract

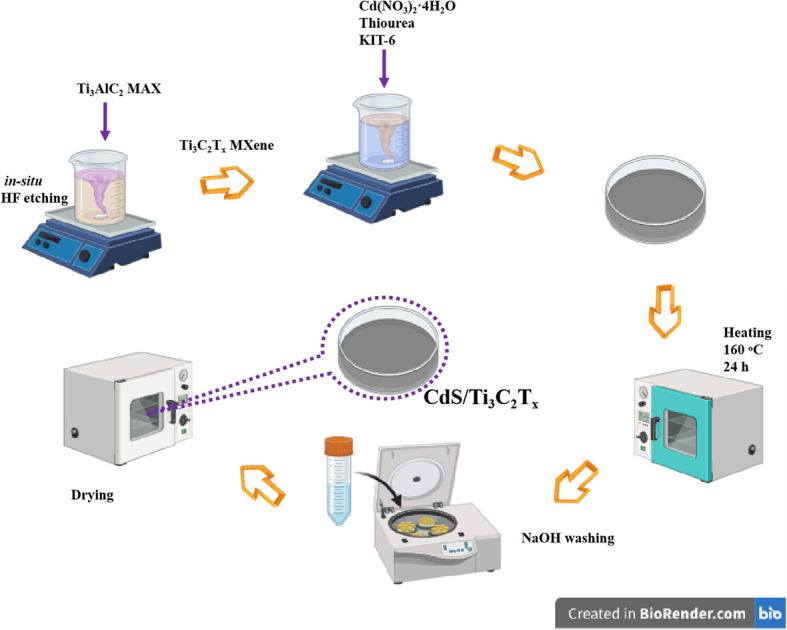

## 1. Introduction

Volatile organic compounds (VOCs) are flammable and poisonous gases. They are found mostly in refineries, mining industries, and our daily lives. Exposure to VOCs by animals and humans can affect the respiratory system. Ethyl alcohol (ethanol) is popularly utilized as a organic solvent in cosmetics, and chemical laboratories.^1–4^ Ethyl alcohol is a well-known VOC and can be hazardous to consciousness upon heavy exposure, causing dizziness, vomiting, and nausea. Additionally, extreme leakage of ethyl alcohol could cause fires and explosions because of its high flammability. Most detection sensors in the market are costly and consume a lot of power because of the sensor material and design.^5,6^ Consequently, developing a willingly accessible and low-cost gas detector with a limit of detection towards that of ethyl alcohol is crucial for environmental monitoring. Resistive gas sensors are attractive thanks to their swift and specific determination of toxic gases compared with heavyweight and nonportable systems such as liquid chromatography-mass spectrometry, flue gas analyzers, and gas chromatography-mass spectrometry.^7,8^

Since the first exploration of two-dimensional materials in 2004, graphene has garnered rigorous research curiosity owing to its chemical and physical properties.^9^ Besides, MXenes could be used as gas sensors owing to their surface functional groups and metallic properties. These multilayer MXenes have a universal formulation of M_*n*+1_X_*n*_T_*z*_ (*n* = 1–3) whereby M denotes early transition metals (Mo, Ti, V, Nb, Cr), X is nitrogen and/or carbon, and T_*z*_ represents the termination groups (F, Cl, Br, I, O, OH).^10–14^ In 2017, the first report about gas sensing of Ti_3_C_2_T_*x*_ MXene was published by Lee *et al.*,^15^ which displayed p-type sensing properties toward ammonia, acetone, methanol, and ethanol. That report was followed by a speedily increasing number of studies due to their excellent properties, such as electrical conductivity (10^3^–10^4^ S cm^−1^), large surface area, high hydrophilicity, versatile surface chemistry, and high mechanical stability. Interestingly, the surface termination groups of Ti_3_C_2_T_*x*_ MXene offer a hydrophilic surface with a greatly negative zeta potential (−30 to −80 mV), which enables effective processing of a combination of Ti_3_C_2_T_*x*_ MXene with other nanomaterials to form a smart composition.^16–19^

2D MXene nanosheets are readily restacked after a long time, which results in low sensitivity and stability of gas sensors made of pristine MXene.^20^ Furthermore, pure Ti_3_C_2_T_*x*_-MXene gas sensors are unsatisfactory in terms of the limit of detection and selectivity.^21^ In order to overcome these problems, the construction of heterostructures has improved the performance of MXene-based gas sensors. Ti_3_C_2_T_*x*_ MXene is modifiable thanks to its excellent electrical characteristics and its high electronic density in the Fermi energy level. Studies have shown that the construction of heterostructures can effectively increase the number of adsorption sites and improve gas sensing capabilities.^22,23^ The MXene/In_2_O_3_ heterostructure, formed *via* a simple process, disperses In_2_O_3_ on MXene layers, enabling NH_3_ detection with high sensitivity and selectivity at room temperature.^24^ The MXene/SnS_2_ heterojunction sensor exhibits excellent sub-ppm ammonia detection, sensing NH_3_ down to 10 ppb at room temperature.^25^ The room temperature sensor has a crucial role because high temperatures impede its usability in wearable applications. Operating at high temperatures results in increased energy consumption when detecting toxic gases at high concentrations. Furthermore, high-temperature operation compromises the nanostructure of the sensing material (thereby reducing its gas sensing efficiency) but also poses challenges in detecting explosive or flammable gases.^12,26^

Cadmium sulfide (CdS) stands out due to its small bandgap (2.4 eV), efficient absorption of light, and excellent charge separation properties.^27,28^ Additionally, CdS is stable, non-toxic, and safe.^29^ As an important member of the II–VI compound family, CdS has been widely applied in various fields and studies, including sensors, lasers, solar cells, photoelectric detection, biomedical tags, photocatalysis, electroluminescent devices, the hydrogen evolution reaction (HER), and photoconductors.^30–33^ CdS has also been reported in sensor applications for the detection of gases.^34,35^ For example, Giberti *et al.* developed a gas sensor using an as-synthesized CdS thick film.^36^ However, the strong selectivity for alcohols requires a temperature of 300 °C, which is too high for practical applications. The high working temperature, high limit of detection, and poor conductivity of CdS hinder its application in CdS-based gas sensing. Therefore, there is an immediate requirement to develop a CdS-based gas sensor with high selectivity and enhanced responding performance at room temperature. Recently, Guo *et al.* demonstrated that having many active sites can enhance gas sensing properties by boosting the adsorption capacity for the target gas, thus increasing the affinity for gas molecules.^29^ According to our previous research, nanostructured semiconductors with mesoporous, nanomesh, and nanowire structures can provide many active sites.^37–39^ Moreover, semiconductors with narrow energy band gaps (*e.g.*, CdS, CdO) exhibit higher charge carrier concentrations but also lower resistivity, which enhances the adsorption energy of oxidizing gas molecules.^40–42^

It has been reported that CdS/Ti_3_C_2_T_*x*_ can be used in the photocatalytic field. A stable Ti_3_C_2_T_*x*_ MXene/CdS heterojunction catalyst, created *via in situ* growth of CdS on Ti_3_C_2_T_*x*_ MXene, enhances hydrogen production by broadening NIR absorption, accelerating photochemical reactions, enabling efficient electron separation, and reducing carrier recombination.^43^ In another study, it has been demonstrated that grafting MXene with CdS enhances charge separation, optimizes pore structure, and increases surface area, improving photocatalyst-reactant contact.^44^ In the meantime, ultrathin Ti_3_C_2_T_*x*_ can reduce CdS aggregation, forming Schottky junctions that boost photocatalytic activity by improving charge separation and electron transfer.^45^ It also enhances H_2_O_2_ selectivity and enables a high-performance photo-Fenton system. In CdS, the sulfur (S) element exhibits low electronegativity, which facilitates the adsorption of oxygen molecules and their subsequent reaction with reactive sulfur species (S^2−^) to generate a series of ionized oxygen species. Consequently, the activation energy required for the conversion of O_2_ molecules to these ionized oxygen species at CdS sites is lower than that necessary on metal oxide surfaces. As a result, more ionized oxygen species are generated on CdS structures. It is well established that ionized oxygen species have critical roles in gas sensing reactions. Thus, CdS-based sensors may operate at lower temperatures compared with traditional oxide semiconductor materials.^46^ However, the combination of Ti_3_C_2_T_*x*_ MXene and CdS nanomaterials for use as a gas sensor has rarely been reported.

In this research, we synthesized a CdS/Ti_3_C_2_T_*x*_ composite using Ti_3_C_2_T_*x*_ MXene and mesoporous CdS nanomaterials with a large surface area and uniform pore size. KIT-6, an ordered mesoporous silica, was chosen as a template because of its bicontinuous mesoporous structure, which belongs to the cubic *Ia*3*d* space group.^33^ This silica consists of enantiomeric pairs of interpenetrating mesoporous networks, all with uniform pore diameters. The three-dimensional (3D) connectivity of the pores make it an ideal template for creating a self-retaining replica.^27,39^ In addition, the pore diameters can be controlled in the range 4–12 nm by adjusting the synthetic conditions. Overall, the combination of the large pore size and high stability of KIT-6 and 3D interconnected mesopores makes it a highly versatile and effective template for the synthesis of advanced functional materials with “tailored” properties. We examined the VOCs gas-sensing activity of pure Ti_3_C_2_T_*x*_ MXene and its hybrid structure at room temperature. The mesoporous structure significantly enhanced gas sensing performance by providing a high surface area and interconnected pores that facilitated efficient gas adsorption and diffusion. This structure ensured that target gas molecules could readily access the active sensing sites, thereby increasing sensitivity and enabling fast response and recovery times. In addition, the mesoporous framework supported the formation of nanostructures with optimized morphology, thereby improving the stability and reproducibility of sensor performance. These combined effects make mesoporous materials highly effective for gas-sensing applications. The optimized composite specified a response of 31% at 20 ppm at room temperature with outstanding repeatability. We demonstrated that the mesoporous CdS nanostructures covering Ti_3_C_2_T_*x*_ strengthened the surface area but also improved electron transfer between composite and gas molecules. Additionally, the plausible sensing mechanism of CdS/Ti_3_C_2_T_*x*_ sensor was investigated further.

## 2. Experimental

### 2.1. Ti_3_C_2_T_*x*_ MXene preparation

To prepare Ti_3_C_2_T_*x*_ MXene, the *in situ* HF etching method was applied. Briefly, two grams of LiF were spread in 50 mL of HCl solution (5.5 M) under vigorous stirring. Subsequently, two grams of Ti_3_AlC_2_ MAX phase powder (MilliporeSigma) were progressively placed into the solution in an ice bath to mitigate exothermic reactions. The mixture solution was then kept in a bath at 50 °C for 1 day. The final product was separated by vacuum filtration and washed several times using deionized water until the pH reached around 6–7. The multilayered Ti_3_C_2_T_*x*_ MXene was dried in a freeze-dryer before being stored for further experiments.

### 2.2. Synthesis of KIT-6

KIT-6 was produced according to the previously reported soft-template method with some modifications. KIT-6 was synthesized hydrothermally, following a procedure documented in previous studies.^38^ KIT-6 was synthesized by preparing an aqueous solution with a 1 : 1 weight ratio of Pluronic P123 (EO_20_PO_70_EO_20_, MW = 5800) and butanol, maintaining an HCl concentration around 0.5 M at temperatures between 25 and 35 °C. Both tetraethoxysilane (TEOS) and sodium silicate can serve as silica sources. For a typical synthesis using TEOS, we dissolved 6 g of P123 in 217 g of distilled water and added 11.8 g of concentrated HCl (35%). Then, we incorporated 6 g of butanol while stirring at 35 °C. After 1 h of stirring, we added 12.9 g of TEOS at the same temperature. We continued stirring the mixture for 24 h at 35 °C, then heated it for an additional 24 h at 100 °C under static conditions in a sealed polypropylene bottle. The solid product from the hydrothermal treatment was filtered and dried at 100 °C without washing. The template was removed by extracting it in an ethanol solution over 3 days.

### 2.3. Preparation of mesoporous CdS nanomaterials

We dissolved 0.6 g of Cd(NO_3_)_2_·4H_2_O and 0.2 g of thiourea in 15 mL of ethanol at room temperature, then added 0.8 g of the KIT-6 template to the stirring solution. After evaporating the ethanol, we spread the solid mixture in a Petri dish. The mixture was heated at 160 °C for 24 h in an air atmosphere to form the CdS@KIT-6 intermediate. The silica template was removed by treating the mixture with 50 mL of 2 M NaOH aqueous solution, which served as an etching agent, to obtain mesoporous CdS.

### 2.4. Preparation of CdS/Ti_3_C_2_T_*x*_ hybrids

**Fig. 1:**
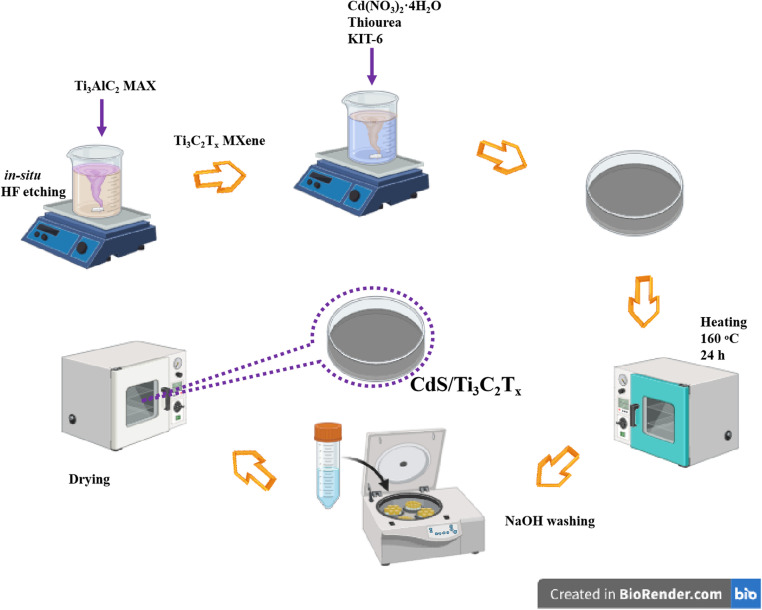
Synthesis of CdS/Ti_3_C_2_T_*x*_ composites (schematic).

We dissolved 0.11 g of Cd(NO_3_)_2_·4H_2_O and 0.03 g of thiourea in 10 mL of ethanol at room temperature, and then added 0.3 g of the KIT-6 template to the stirring mixture. Subsequently, we gradually incorporated the pre-prepared Ti_3_C_2_T_*x*_ MXene (100, 200, or 300 mg) into the solution and stirred vigorously for 15 min. After evaporating the ethanol, we spread the solid mixture in a Petri dish. The mixture was heated at 160 °C for 24 h in an air atmosphere to form the CdS@KIT-6/Ti_3_C_2_T_*x*_ intermediate. The silica template was removed by treating the mixture with 50 mL of 2 M NaOH aqueous solution, which acted as an etching agent, to obtain CdS/Ti_3_C_2_T_*x*_. The final products were named “CT1” (100 mg), “CT2” (200 mg), and “CT3” (300 mg) (Fig. 1). For comparison, pure mesoporous CdS was also prepared under the same conditions, without the addition of Ti_3_C_2_T_*x*_ MXene.

### 2.5. Characterization and measurements

The characterization methods employed in this study were: X-ray diffraction (XRD; Rigaku SmartLab) using Cu Kα radiation to analyze sample phase composition; scanning electron microscopy (SEM; Hitachi S-4700) to observe surface chemical characteristics and morphologies; high-performance X-ray photoelectron spectroscopy (XPS; K-Alpha^+^) for investigating chemical states; and Brunauer–Emmett–Teller (BET) analysis using an ASAP 2020 accelerated surface area and porosimetry system to determine the specific surface areas and pore distributions of the powders. The optical properties of synthesized samples were recorded using UV-vis diffuse reflectance spectroscopy (Jasco V-770).

### 2.6. Gas sensor experiments

The toxic gas sensing activity of the CdS/Ti_3_C_2_T_*x*_ heterostructure gas sensor was recorded using a homemade system. The as-synthesized samples were dispersed in deionized water at a concentration of 1 mg mL^−1^ and then drop-coated onto a silicon wafer (1 × 1 cm) with a thickness of approximately 8–10 µm. The sensor wafer was dried overnight to evaporate the solvent and subsequently placed within a stainless-steel chamber connected to a source measure unit (Keysight 34465A). The relative humidity of the gas testing system was calibrated by adjusting the parameters in a humidity meter. The gas concentration was calibrated to the desired level using a mass flow controller and mass flow meter (MFC/MFM) by adjusting the flow rates of synthetic air and the target gas. The diluted gas mixture was subsequently injected into the chamber after thorough mixing. The response of the CdS/Ti_3_C_2_T_*x*_ heterostructure was calculated as the alteration in the relative resistance in the target gas (*R*_gas_) compared with that in synthetic air (*R*_air_) before and after target gas injection, as follows:
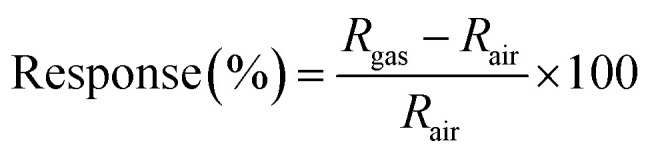


## 3. Results and discussion

### 3.1. Morphological and component characterization

**Fig. 2:**
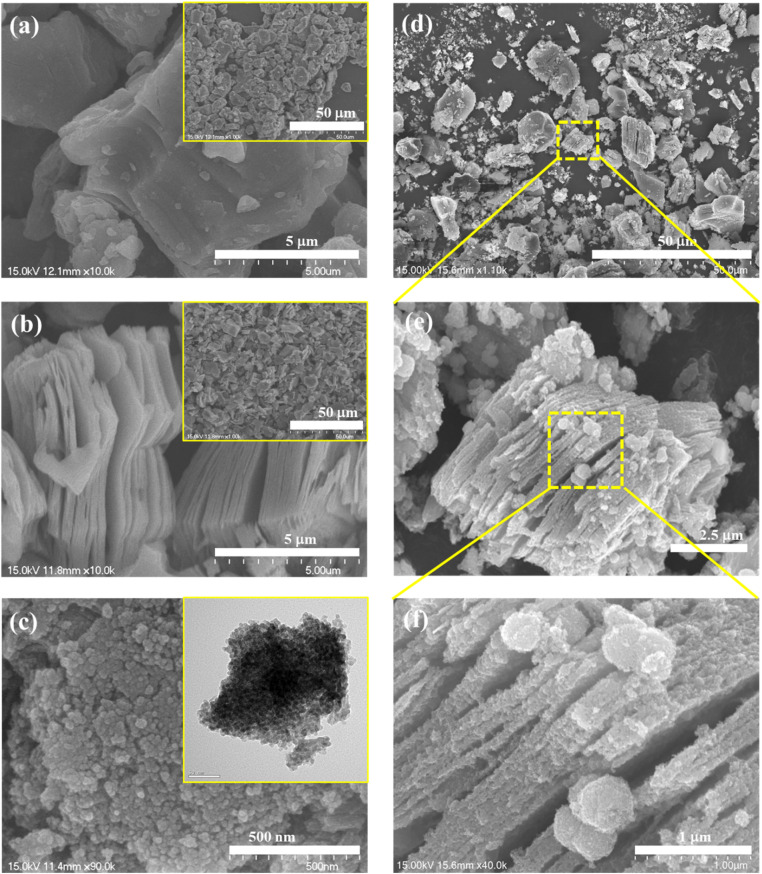
SEM images of (a) pristine Ti_3_AlC_2_, (b) Ti_3_C_2_T_*x*_ MXene, (c) CdS (the inset figure is the TEM of CdS) and (d–f) the CdS/Ti_3_C_2_T_*x*_ heterostructure.

Fig. 2 displays the FE-SEM images of pure Ti_3_AlC_2_, Ti_3_C_2_T_*x*_, CdS and the CdS/Ti_3_C_2_T_*x*_ heterostructure. As shown in Fig. 2a, commercial Ti_3_AlC_2_, had stacked, laminated and compact structures. After etching with HF acid, the Ti_3_C_2_T_*x*_ MXene with layered and accordion-like structures was observed in Fig. 2b. Pure CdS showed a spherical granule-like structure with and average diameter of 20 nm (Fig. 2c). With abundant hydrophilic groups, CdS nanoparticles covered readily on the superficial of Ti_3_C_2_T_*x*_ MXene, further confirming the combination of the intimate interfacial interaction between Ti_3_C_2_T_*x*_ MXene and CdS (Fig. 2d–f).

**Fig. 3:**
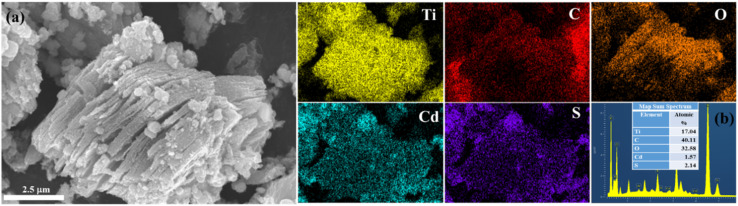
(a) SEM images and (b) SEM-EDX elemental mapping of the CdS/Ti_3_C_2_T_*x*_ heterostructure.

Moreover, the results of EDS elemental mapping revealed the uniform distribution of Cd, S, Ti, C, and O elements in the CdS/Ti_3_C_2_T_*x*_ heterostructure. As shown in Fig. 3a, numerous CdS NPs were decorated on the Ti_3_C_2_T_*x*_ MXene and a few were agglomerated on the Ti_3_C_2_T_*x*_ MXene. The direct close interaction between CdS and Ti_3_C_2_T_*x*_ MXene boosted the fast transfer and migration of generated electrons. As presented in Fig. 3b, a higher oxygen atom content (32.6% atomic%) than that in pristine Ti_3_C_2_T_*x*_ MXene (14%) was obtained, resulting in more oxygen termination groups, and partial formation of TiO_2_ during the hydrothermal reaction.

To understand the optical properties, we also obtained the energy band gap of typical samples. Fig. S1† displays the UV-vis diffuse reflectance spectra of selected samples over a wavelength range of 300–800 nm. Notably, an absorption edge peak of CdS was observed at ∼500 nm (Fig. S1a†). In comparison with pristine CdS, the optical absorption edge of the composite exhibited a distinct red shift to ∼550 nm. This 550 nm absorption edge arose from the interband electronic transitions in CdS, indicating that CdS had been decorated and coupled within the composite.^47^ As shown in Fig. S1b and d,† the estimated energy band gap (Eg) of pure CdS and the composite was 2.14 eV and 1.58 eV, respectively. A composite with a narrow band gap allows for easier excitation of electrons from the valence band (VB) to the conduction band (CB) with lower energy. This implies that less energy is required to trigger the electronic response of a composite to external stimuli, such as gas adsorption, potentially enhancing the sensitivity of the sensor due to the increased responsiveness to environmental changes of the material.^40–42^

### 3.2. Structural properties

**Fig. 4:**
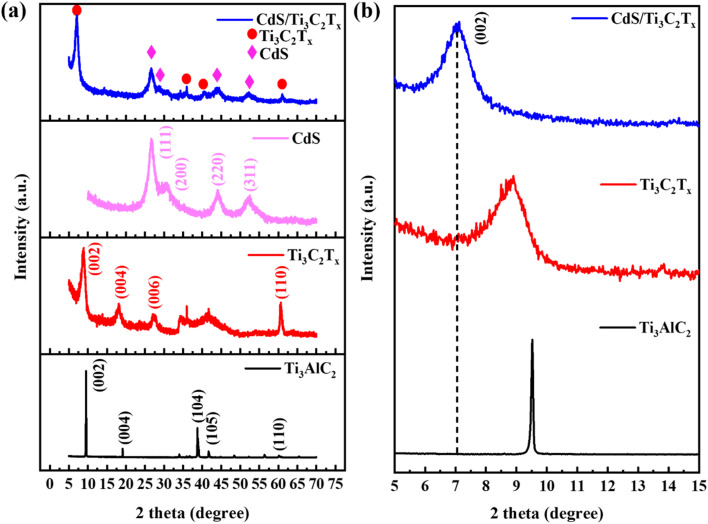
(a) High angle and (b) low angle XRD patterns of Ti_3_AlC_2_ powder, Ti_3_C_2_T_*x*_ MXene, mesoporous CdS nanoparticle, and the CdS/Ti_3_C_2_T_*x*_ heterostructure.

Fig. 4 illustrates the detailed crystalline phase structures measured by XRD. Fig. 4a clearly shows that the (002) and (004) peaks of the as-prepared Ti_3_C_2_T_*x*_ MXene were shifted to smaller angles and broadened compared with those of Ti_3_AlC_2_ powder, indicating the formation of a laminated microstructure after HF etching.^48^ The diffraction peak intensity of Ti_3_C_2_T_*x*_ was significantly lower than that of Ti_3_AlC_2_. This disparity may have arisen from differences in the distribution of carbon atoms within the crystal structure and the crystal symmetry of both materials. Additionally, diffraction peaks of the original Ti_3_AlC_2_ MAX phase between 35° and 40° vanished, which suggested that Al atoms had been removed. This result also showed that our synthesized Ti_3_C_2_T_*x*_ had high purity, which is in good agreement with previous reports.^49^ In the CdS/Ti_3_C_2_T_*x*_ MXene heterostructure, the diffraction peak of both Ti_3_C_2_T_*x*_ MXene and mesoporous CdS nanoparticles could be observed. The clear diffraction peaks at 2*θ* = 26.6°, 30.4°, 44.1°, and 52.5° corresponded to the phases (111), (200), (220) together with (311) planes, and confirmed the existence of lattice plates of cubic zinc blended crystal structure (PDF 01-075-0581).^50^ In particular, in the CdS/Ti_3_C_2_T_*x*_ MXene heterostructure, the (002) peak was notably broadened and shifted to a lower angle compared with that in the pure Ti_3_C_2_T_*x*_ MXene material, indicating an enlarged layer spacing (Fig. 4b).

**Fig. 5:**
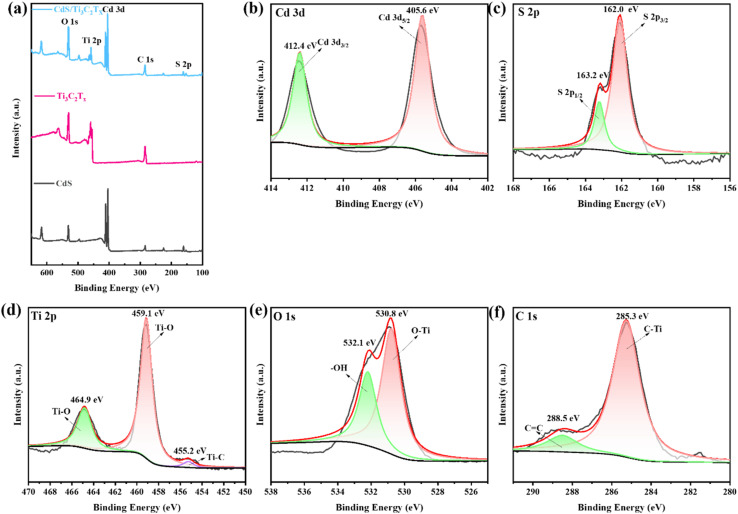
(a) Survey spectra of the mesoporous CdS nanoparticle, Ti_3_C_2_T_*x*_ MXene, and CdS/Ti_3_C_2_T_*x*_ heterostructure. XPS spectra of the CdS/Ti_3_C_2_T_*x*_ heterostructure Cd 3d (b), S 2p (c), Ti 2p (d), O 1s (e), and C 1s (f).

XPS spectra were used to analyze the overall XPS spectra chemical state of the surface as well as the composition of the CdS/Ti_3_C_2_T_*x*_ heterostructure. The survey spectra of Ti_3_C_2_T_*x*_ MXene, mesoporous CdS nanoparticle, and CdS/Ti_3_C_2_T_*x*_ heterostructure are depicted in Fig. 5a. In addition, there were five main chemical elements on the surface, which corresponded to the elements of Cd, S, Ti, O, and C. The highly resolved spectra of the Cd 3d, S 2p, Ti 2p, O 1s and C 1s core levels for the CdS/Ti_3_C_2_T_*x*_ heterostructure are displayed in Fig. 5b–f. The Cd-3d characteristic peaks located at 405.6 and 412.4 eV belonged to the Cd 3d_5/2_ and Cd 3d_3/2_ orbitals (Fig. 5b), respectively, indicating the presence of Cd ions in the +2 oxidation state.^51,52^ Two significant peaks located at 162.0 and 163.2 eV, which were equal to the binding energy of S 2p_3/2_ and S 2p_1/2_ (Fig. 5c), showed the presence of S–S bonding in CdS.^44,53^ The Ti 2p binding energy exhibited three distinct peaks centered at 455.2 eV, 459.1 eV, and 464.9 eV, which corresponded to Ti–C and Ti–O bonds, respectively, within the Ti 2p domain (Fig. 3d). As shown in Fig. 3e, the O 1s spectrum exhibited two main peaks at 530.8 eV and 532.1 eV, which were attributed to hydroxyl/oxygen-terminated surfaces and *in situ* oxidized TiO_2_.^54^ The spectrum of the C 1s peak showed two distinct peaks at 285.3 eV and 288.5 eV, which corresponded to C=C and C–Ti bonds on the surface, respectively, as depicted in Fig. 5f.^55,56^ The characterization data further indicated fabrication of the CdS/Ti_3_C_2_T_*x*_ heterostructure.

**Fig. 6:**
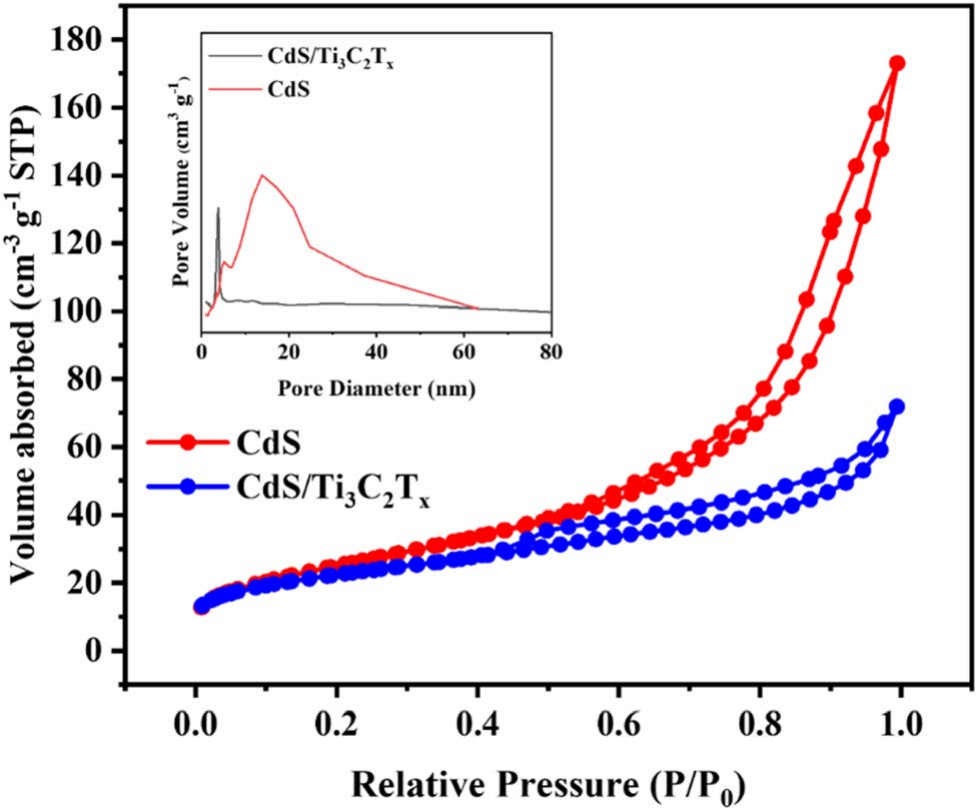
Nitrogen adsorption–desorption isotherms were conducted for Ti_3_C_2_T_*x*_ MXene, mesoporous CdS nanoparticles, and the CdS/Ti_3_C_2_T_*x*_ heterostructure. Pore-size distributions are depicted in the inset.

The nitrogen adsorption–desorption isotherms of the as-prepared materials were employed to evaluate the impact of mesoporous CdS nanoparticles on the surface area of Ti_3_C_2_T_*x*_ MXene. This evaluation aimed to assess potential improvements in gas sensing performance in terms of gas recovery and response time. As illustrated in Fig. 6, the nitrogen sorption isotherms of CdS, and CdS/Ti_3_C_2_T_*x*_ samples indicated the pore characteristics of these samples with respect to a type-IV isotherm with a long and narrow hysteresis loop, suggesting the presence of uniform mesoporosity.^57^ The BET surface area of the mesoporous CdS and the CdS/Ti_3_C_2_T_*x*_ heterostructure was estimated to be 81.1 and 78.4 m^2^ g^−1^, respectively. In addition, the average pore width of the mesoporous CdS, and CdS/Ti_3_C_2_T_*x*_ heterostructure was 8.9, and 6.7 nm, respectively (inset of Fig. 6). These results showed that, after the preparation of mesoporous CdS and Ti_3_C_2_T_*x*_ MXene as heterojunction materials, a larger pore size and higher specific surface area were observed, which increased the number of active sites and facilitated electron transport.

### 3.3. Target gas sensing performance and mechanism

**Fig. 7:**
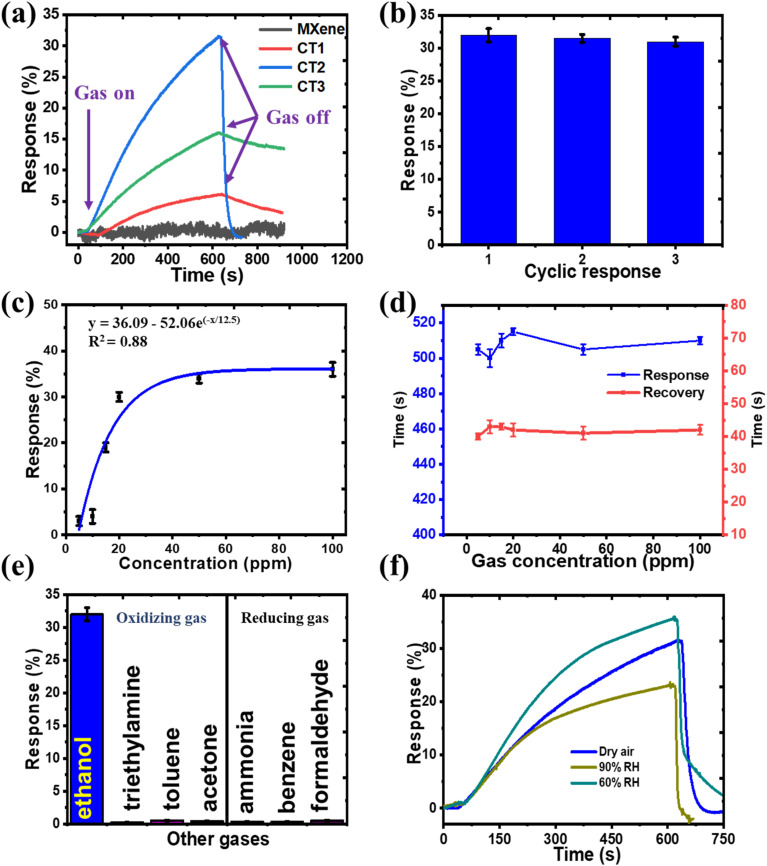
(a) The response/recovery properties of pristine Ti_3_C_2_T_*x*_ MXene, and prepared CdS/Ti_3_C_2_T_*x*_-based sensors, toward ethanol molecules (20 ppm). (b) Repeatability curve and (c) Langmuir isotherm curve of CT2 responses against different ethanol concentrations, and (d) response and recovery time. Response of the CT2 sensor to (e) several gases (f) at various levels of relative humidity.

The gas sensing activity of the as-prepared CdS/Ti_3_C_2_T_*x*_ heterostructure-based sensors was recorded at room temperature by analyzing the resistance variation in target gases and air. First, the synthesis air was used to flow the chamber and obtain a stable condition. Fig. 7 displays the gas response of prepared CdS/Ti_3_C_2_T_*x*_-based sensors to different target gas molecules. In Fig. 7a, we analyzed the responses of the as-synthesized CT1, CT2, CT3, and pure Ti_3_C_2_T_*x*_ MXene to 20 ppm ethanol gas. The CdS/Ti_3_C_2_T_*x*_ heterostructure displayed an obvious reaction to ethanol, while Ti_3_C_2_T_*x*_ MXene had no signal as metallic properties. The CT2 heterostructure displayed the highest gas response as compared with that of CT1, CT3, and pure Ti_3_C_2_T_*x*_. The repeatability performance of CT2 sensors to 20 ppm ethanol after three cycles was also tested under the same parameter. As revealed in Fig. 7b, the gas responses of the CT2 sample varied slightly and had a similar tendency, which displayed reproducible results.

Fig. 7c presents the sensor response curves of CT2 against ethanol concentrations ranging from 5 to 100 ppm. All response signals increased with an increase in ethanol concentration, demonstrating a good correlation. The calculated linear regression coefficient (*R*^2^) was ∼0.88, indicating that CT2 was logarithmic in detecting ethanol molecules within this concentration range. Fig. 7d illustrates the response and recovery times as a function of gas concentration, with error bars included. The response time was ∼507 s, compared with a recovery time of 41 s. The relatively long response time could be attributed to the need for ethanol gas to diffuse and undergo subsequent oxidation during sensing. The response time of a sensor was influenced by the number of available reactive sites, which required time to reach the optimal configuration for gas-sensing reactions. In contrast, a faster recovery time was attributed to the porous structure and rapid electron transport of the material, which provided a large surface area for diffusion and facilitated the removal of ethanol molecules under 100% airflow. This phenomenon has also been observed in previous studies.^49,58^

To further confirm the selectivity of the CdS/Ti_3_C_2_T_*x*_ heterostructure, the signal histograms of the optimized CT2 sensor for a variety of target gases to 20 ppm are presented in Fig. 7e. The signal responses to different target gases (acetone, toluene, formaldehyde, ammonia, benzene, triethylamine) were negligible. Only the CdS/Ti_3_C_2_T_*x*_ heterostructure displayed a response to ethanol. These results further confirmed the selectivity of the CdS/Ti_3_C_2_T_*x*_ sensor to ethanol. Several factors may have contributed to the observed selectivity. Ethanol possesses a hydroxyl (–OH) group and a hydrocarbon chain. Although it is not perfectly symmetrical, it exhibits some symmetry that can influence its interaction with surfaces. The presence of methyl groups affects polarity and induces dipole moments. Ethanol, which primarily contains single bonds, has a lower molecular mass and volatile nature, whereas others contain carbonyl or aromatic structures with double bonds. In contrast, toluene and benzene are nonpolar compounds, resulting in minimal interaction with the sensor surface.^59^ On the other hand, the greater selectivity of the CdS/Ti_3_C_2_T_*x*_ composite can be explained *via* the different unoccupied molecule orbit (LUMO) energies of these gas molecules. The high value of the LUMO energy indicates that a significant amount of energy is required for the interaction between the sensor material and target gas.^60,61^ The LUMO energy of ethanol (0.12 eV) is the lowest among the gases stated above, which is better for electrons and the reaction between the composite and ethanol molecules.^46^ Thus, the CdS/Ti_3_C_2_T_*x*_ composite had excellent selectivity towards ethanol at ambient atmosphere.

Fig. 7f illustrates the response curve of the CdS/Ti_3_C_2_T_*x*_ composite to ethanol (20 ppm) at various levels of relative humidity. As the humidity increases, the hydroxyl groups in H_2_O molecules exhibit weak electron-withdrawing behavior, interacting with electrons from the sensor material and enhancing electrical conductivity, which results in an improved response. However, a further increase in humidity leads to a decrease in response, which can be attributed to the H_2_O poisoning effect. This phenomenon occurs if a significant number of H_2_O molecules adsorb onto the material, obstructing the available adsorption sites for ethanol.^62^

Moreover, the ethanol sensing response of our CdS/Ti_3_C_2_T_*x*_ heterostructures was compared with different MXene composite-based gas sensors: CdS nanostructures, PPy@Ti_3_C_2_T_*x*_, W_18_O_49_@Ti_3_C_2_T_*x*_, MoO_3_@Ti_3_C_2_T_*x*_, and Co_3_O_4_@Ti_3_C_2_T_*x*_ composites. As shown in Table 1, the optimized CT2 did not exhibit the best ethanol sensing response, but it was a promising material for ethanol detection at room temperature.

**Table 1:** Ethanol molecules-sensing performance of previous Ti_3_C_2_T_*x*_-based or CdS-based gas sensors

Material	Temp. (°C)	Concentration (ppm)	Response (%)	Ref.
PPy@Ti_3_C_2_T_*x*_	RT	20	16	63
CdS film	250	5	10	36
CdS hierarchical microspheres	200	10	8	64
0.5 at% CuO–CdS	160	100	7	65
CdS quantum dot	200	1000	0	66
W_18_O_49_@Ti_3_C_2_T_*x*_	300	20	1.67	67
MoO_3_@Ti_3_C_2_T_*x*_	100	20	39	68
Co_3_O_4_@Ti_3_C_2_T_*x*_	200	50	11	69
CdS/Ti_3_C_2_T_*x*_	RT	20	31	This work

**Fig. 8:**
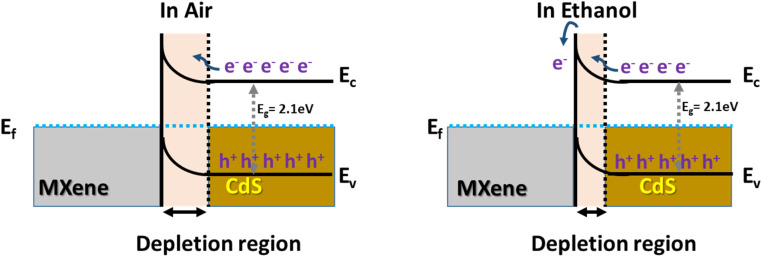
The sensing mechanism of ethanol over the CdS/Ti_3_C_2_T_*x*_ composite.

The improved ethanol sensor mechanism of the CdS/Ti_3_C_2_T_*x*_ composite is displayed schematically in Fig. 8. The p–n junction interface in the composite was formed due to the difference in work functions between n-type CdS (3.2 eV) and p-type Ti_3_C_2_T_*x*_ MXene (5.9 eV).^70^ As a result, electrons from CdS tend to migrate to the MXene interface to form a depletion layer. When the CdS/Ti_3_C_2_T_*x*_ composite is exposed to an air atmosphere, O_2_ molecules are chemically adsorbed and captured on the surface of the sensor material. In particular, the oxygen molecules were converted into ionized adsorbed oxygen (O^−^, O_2_^−^, and O^2−^). When the CdS/Ti_3_C_2_T_*x*_ composite was in contact with and exposed to ethanol molecules, the chemical interaction of ethanol molecules with the ionized adsorbed oxygen could release the generated electron back to the sensor material, resulting in a thinner depletion layer, reduced hole concentration, and increased the resistance of the CdS/Ti_3_C_2_T_*x*_ composite.^64,71–74^ This sophisticated route can be explained by the following equation:O_2_ (g) → O_2_ (ads)O_2_ (ads) + e^−^ → O_2_^−^ (ads)O_2_^−^ (ads) + e^−^ → 2O_2_^−^ (ads)C_2_H_5_OH (gas) + 6O_2_^−^ → CH_3_CHO + 3H_2_O + 12e^−^

## 4. Conclusions

We synthesized VOCs sensors *via* combining CdS nanostructures with Ti_3_C_2_T_*x*_ MXene using a simple method. The as-obtained CdS/Ti_3_C_2_T_*x*_ heterostructures sensor showed a superior sensing activity in the enhanced detection of ethanol gas at room temperature. The CdS/Ti_3_C_2_T_*x*_ heterostructure presented a good response of 31% towards 20 ppm ethanol, which was superior to that of pure MXene. Moreover, the optimized CdS/Ti_3_C_2_T_*x*_ heterostructure presented excellent selectivity with fast recovery time to ethanol molecules. The combination effect of CdS and metallic Ti_3_C_2_T_*x*_ in the composite had a crucial role in ethanol sensing performance. Thus, the optimized CdS/Ti_3_C_2_T_*x*_ heterostructure provides a potential composite to synthesize and improve superior ethanol sensors.

## Data availability

The data supporting this article have been included as part of the ESI.†

## Conflicts of interest

There are no conflicts to declare.

## Supplementary Material

NA-OLF-D4NA00927D-s001
