# Supplementary material for: Highly selective ethanol gas sensor based on CdS/Ti3C2Tx MXene composites
Source: Nanoscale Adv. 2025 Jan 8;7(5):1452–63. doi: 10.1039/d4na00927d (PMC11758100; doi:10.1039/d4na00927d)
Supplement: NA-007-D4NA00927D-s001 [file NA-007-D4NA00927D-s001.pdf]

Supplementary Information (SI) for Nanoscale Advances.
This journal is © The Royal Society of Chemistry 2025

Supporting Information

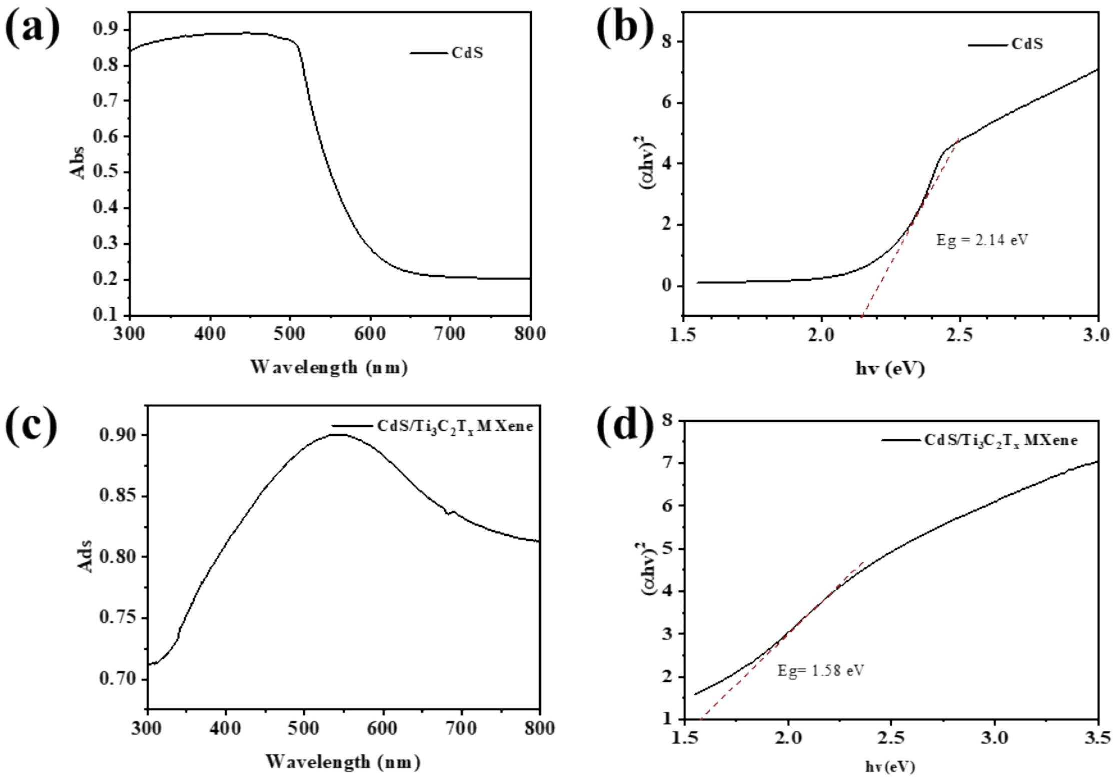

Figure S1. UV–Vis DRS (a, c) and Tauc plots (b, d) of CdS and CT2 heterostructure, respectively.
